# Understanding tobacco and e-cigarette use among university students: a cross-sectional study exploring nicotine dependence, quit intentions, and awareness of cessation support services

**DOI:** 10.1186/s12889-025-24911-6

**Published:** 2025-10-27

**Authors:** Kamer Billur Yücel Özden, Ayşe Gülsen Ceyhun Peker

**Affiliations:** Department of Family Medicine, Faculty of Medicine, Ankara University, Ankara, Turkey

**Keywords:** Tobacco use, Smoking cessation, Electronic cigarette, Nicotine dependence

## Abstract

**Objective:**

While tobacco addiction remains a preventable global public health issue, increasing concern surrounds the growing use of electronic cigarettes(e-cigarette) among young people. This study aimed to investigate the prevalence of tobacco and e-cigarette use, students’ willingness to quit, their awareness of available professional counselling support, and their knowledge of e-cigarettes.

**Methods:**

This cross-sectional study was conducted between March and June 2024 at a public university in Ankara, Turkey with participation of 1144 students. Stratified and cluster sampling methods were used. Data were collected via face-to-face questionnaires assessing sociodemographic characteristics, tobacco use behaviours, attempts to quit, the Fagerstrom Test for Nicotine Dependence, and 14 e-cigarette knowledge statements. Informational leaflets on cessation support and e-cigarette harms were provided to participants following the survey.

**Results:**

Overall, 40.6% reported current use of any tobacco product and 24.3% reported e-cigarette use; among cigarette smokers, 74.4% had low/very low dependence (median 2 pack-years). Although 72.8% wished to quit, only 36.9% knew how to access medical counselling and 22.3% had received quit advice in the past year. In multivariable analyses, higher academic year, male gender, and lower perceived economic status were associated with greater odds of tobacco use, while female gender and studying in the Faculty of Medicine were associated with lower odds of tobacco use. E-cigarette use was primarily motivated by flavour, curiosity, and, to a lesser extent, smoking cessation.

**Conclusion:**

Tobacco product use remains widespread among university students, with a growing preference for new-generation products such as e-cigarettes. However, low nicotine dependence levels and high willingness to quit represent a valuable intervention opportunity. Comprehensive university-based programs focused on prevention, awareness, and smoking cessation support are essential to promote healthy lifestyle behaviours in young adults.

**Trial registration:**

Not Applicable.

**Supplementary Information:**

The online version contains supplementary material available at 10.1186/s12889-025-24911-6.

## Background

Tobacco addiction is a preventable global public health problem that causes the death of millions of people each year [1]. According to the World Health Organization (WHO), there are four main modifiable behavioural risk factors for the prevention of non-communicable diseases, and tobacco addiction is one of them [2]. In this context, tobacco control emphasizes proactive measures aimed at preventing individuals from ever initiating its use. Preventing addiction is a much more effective, sustainable, and socially beneficial approach compared to cessation efforts that are undertaken after addiction has developed and that generate significant health and economic costs.

According to advanced analyses of the Global Burden of Disease Study, 82.6% of tobacco users initiate use between the ages of 14 and 25, and the likelihood of a non-addicted individual developing tobacco dependence after this age range is significantly low [3]. Therefore, adolescents and university-aged young adults constitute the primary risk group that must be protected. During this age period, ongoing neurobiological development, peer influence, and the search for social acceptance make young people particularly vulnerable to nicotine addiction [4]. When the influence of the social environment is combined with the tobacco industry’s marketing strategies for e-cigarettes—featuring attractive flavours and product designs—it becomes easier for young individuals to start using nicotine products. Exposure to nicotine during adolescence and early adulthood increases the risk of addiction and negatively affects brain development—which continues until around the mid-twenties —leading to long-term neurodevelopmental impairment [5, 6]. Therefore, implementing strategies to limit youth exposure to tobacco products remains a critical public health priority.

Despite the tobacco industry’s claims that e-cigarettes are ‘less harmful’ or help with smoking cessation, these products frequently act as a gateway to tobacco use, particularly among adolescents and young adults. Studies have shown that young people who use e-cigarettes are significantly more likely to start smoking conventional cigarettes in the following years [7–9]. In response to this growing concern, the WHO issued an urgent call to action in December 2023, emphasizing the need to safeguard young people from these products [10].

Following Turkey’s ratification of the WHO Framework Convention on Tobacco Control (FCTC) in 2004, a National Action Plan covering the years 2008–2012 was developed. As part of this plan, the Law on the Prevention of the Harms of Tobacco Products (Law No. 4207) was amended to expand smoke-free regulations. Consequently, smoking was banned in all enclosed public spaces, except for prisons, psychiatric hospitals, and elderly care facilities. In 2013, a legal amendment extended the scope of Law No. 4207 to include products that do not contain tobacco but are used in a similar manner to tobacco products. Furthermore, a Presidential Decree issued in 2020 prohibited the importation of electronic nicotine delivery systems, including e-cigarettes and similar devices (Presidential Decree on the Importation of Electronic Cigarettes and Similar Devices, No. 2149, 23.02.2020.) [11].

Within the scope of tobacco control policies, smoking cessation clinics have been established in primary care centres and hospitals across Turkey. These clinics provide individuals with both pharmacotherapy (such as nicotine replacement therapies, bupropion, or varenicline) and behavioural counselling. In addition, the Ministry of Health established a Smoking Cessation Advice Line (ALO 171), which operates on a 24/7 basis, offering counselling services and directing individuals to appropriate cessation clinics. Studies conducted in smoking cessation outpatient clinics in Turkey have shown that counselling, either alone or combined with pharmacological treatment, leads to substantial success in smoking cessation. Alongside these clinical services, referrals made through the national quit line facilitate easier access to cessation support for individuals willing to quit smoking [12].

Field-based research aimed at understanding the behavioural patterns of the youth population plays a critical role in tobacco control. According to the most recent official data on young people of university age in Turkey, the 2016 Global Adult Tobacco Survey (GATS) reported that 31.9% of individuals aged 15–24 used tobacco products [13]. A more recent survey conducted by the Ministry of Health— the 2023 Turkey Household Health Survey— found that the tobacco use rate among individuals aged 15–29 was 33% [14]. However, these national surveys do not provide numerical data on electronic nicotine delivery systems (such as e-cigarettes and similar products). Even though the sale of these products is not legally permitted in the country, their use is becoming increasingly widespread, and studies on the topic remain limited to local research. For instance, a study conducted in İzmir reported that 19% of participants had tried e-cigarettes at least once [15], while another study involving 2477 university students found the current use of e-cigarettes to be 2.1% [16].

This study aimed to investigate the prevalence of tobacco product use, willingness to quit, methods used for cessation, and knowledge about e-cigarettes among university students. Additionally, through informational leaflets distributed to students after the survey, the study seeks to raise awareness among those who wish to quit tobacco use and to provide information on the harms of e-cigarettes.

## Materials and methods

This cross-sectional study was conducted from March to June 2024 at a public university located in Ankara, the capital city of Turkey, which attracts a broad and heterogeneous student population from various regions of the country.

The study population consisted of a public university with approximately 60,000 students. The sample size was calculated using the OpenEpi software. Faculties admitting students based on various academic score types were selected for stratified sampling. Subsequently, cluster sampling was employed to distribute the survey to students in designated classrooms during lecture breaks. Classroom selection was carried out in coordination with faculty administrations, considering course schedules and academic term structures to ensure efficient time management and maximum participation. The survey was administered to undergraduate students, including international students. All students present in the selected classrooms who were willing to participate were included in the study; those who did not wish to participate were not surveyed, and no additional exclusion criteria were applied. Nearly all students in the selected classes agreed to participate. According to the Global Adult Tobacco Survey (GATS), the prevalence of tobacco product use among individuals aged 15–24 in Turkey is 31.9%. Based on this data, with a 95% confidence interval and a 6% margin of error, the target number of students to be reached was determined as follows: 214 from the Faculty of Medicine, 189 from the Faculty of Engineering, 215 from the Faculty of Law, and 227 from the Faculty of Languages, History and Geography.

The data collection form included items assessing students’ sociodemographic characteristics, tobacco use habits among those who use tobacco products, their willingness and attempts to quit, and questions evaluating knowledge about e-cigarettes (prepared using national and international authoritative sources). In addition, the Fagerstrom Test for Nicotine Dependence was used to assess the level of nicotine addiction among cigarette smokers. Questions about e-cigarettes were not directed to students who did not use tobacco products (including e-cigarettes), to prevent misleading or irrelevant responses. The questionnaire used in this study was prepared by the research team using national and international authoritative sources. An English version of the questionnaire is provided as Supplementary File 1. After the survey, students were provided with an informational leaflet that included correct answers to the e-cigarette-related questions in the questionnaire, the benefits of quitting tobacco use, as well as information about the national smoking cessation counselling hotline and smoking cessation clinics.

Administrative approval for the study was obtained from the Dean’s offices of the relevant faculties, and ethical approval was granted by the Ankara University Human Research Ethics Committee. Statistical analyses were performed using IBM SPSS version 29 (Statistical Package for the Social Sciences; Chicago, Illinois, USA). Descriptive variables were presented as number and percentage, mean ± standard deviation, or median (1st – 3rd Quartiles), depending on the distribution of the data. For comparisons between groups, the Chi-square and Kruskal–Wallis tests were used based on the distribution characteristics of the variables. To identify independent factors associated with any tobacco product use, a multivariate logistic regression analysis was conducted. In the multivariable logistic regression model, age, faculty, academic year, gender, perceived economic status, and residence status were included as independent variables, while current use of any tobacco product was defined as the dependent variable. A Type I error rate of 0.05 was accepted for all statistical analyses.

## Results

**Fig. 1:**
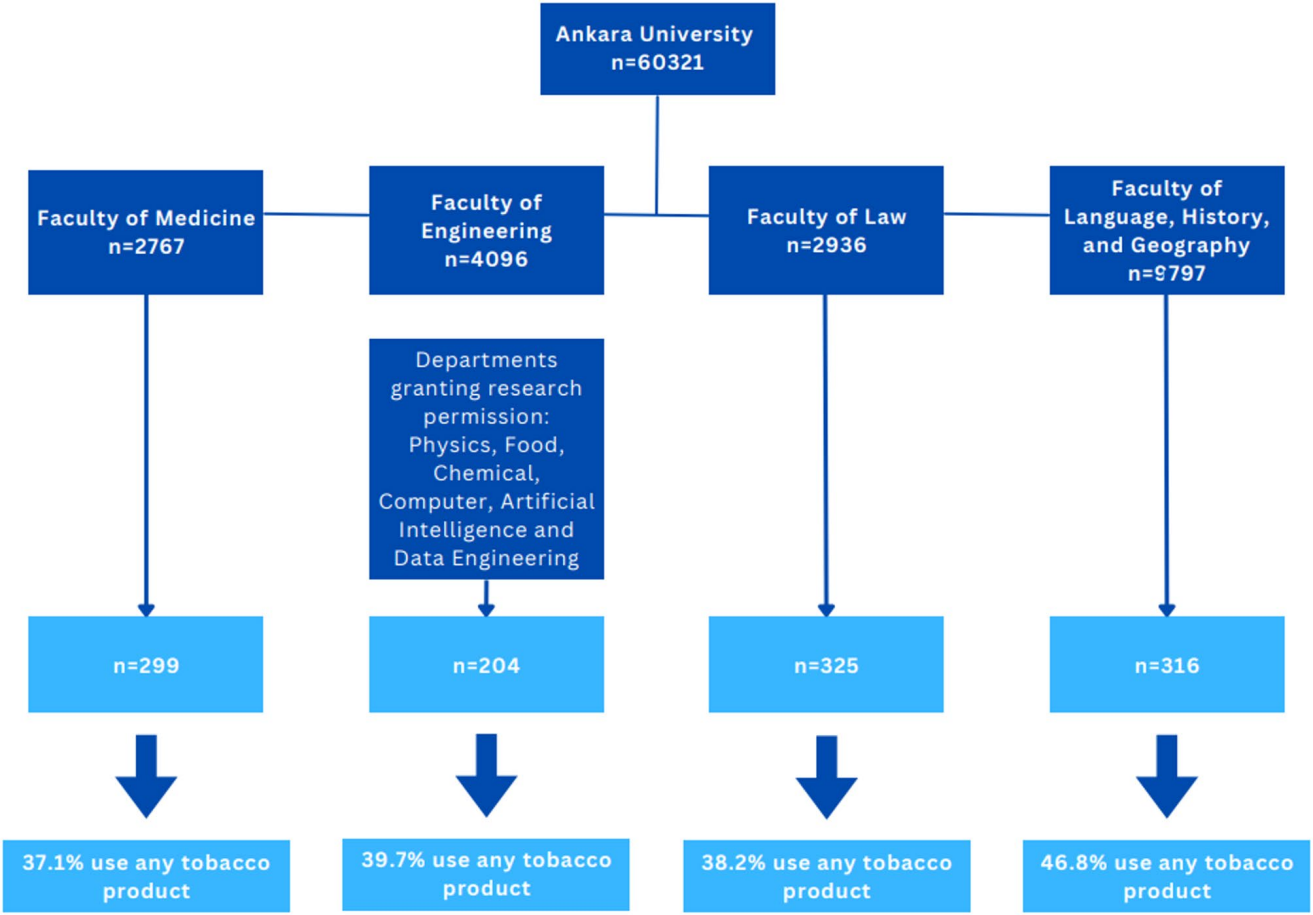
Flowchart of the study. Any tobacco product: cigarettes, e-cigarettes waterpipe(hookah) roll-your-own tobacco, cigar, pipe

The study was completed with the participation of 1,144 students: 299 from the Faculty of Medicine, 204 from the Faculty of Engineering, 325 from the Faculty of Law, and 316 from the Faculty of Languages, History and Geography. The study flow diagram is presented in Fig. 1.

The descriptive characteristics of the participants are presented in Table 1. The majority of the participants were female (55.4%), and students’ mean age was 22.0 ± 3.7 years. Participants’ tobacco use habits and their distribution by faculty are presented in Table 2. Among participants, 40.6% (*n* = 464) reported current tobacco use, while 53.3% (*n* = 610) had never used any tobacco product. Cigarettes were the most common (39.1%, *n* = 447), followed by e- cigarettes (24.3%, *n* = 278), waterpipes (7.8%, *n* = 89), roll-your-own tobacco (7.7%, *n* = 88), and other tobacco products (1.4%, *n* = 16), including cigars, pipes, and heated tobacco products. 96% of the participants who used e-cigarettes also smoked conventional cigarettes. Among cigarette users (*n* = 431), the median cigarette consumption was 2 pack-years (1st – 3rd Quartile: 1–4). According to the Fagerström Test for Nicotine Dependence, nicotine addiction levels among cigarette smokers were as follows: 5.1% (*n* = 22) were very highly dependent, 11.8% (*n* = 51) were highly, 8.6% (*n* = 37) were moderately, 22.7% (*n* = 98) were low, 51.7% (*n* = 223) were very low dependent.

**Table 1:** Tobacco product use by academic year, gender, residential status, and self-perceived economic status

	Any Tobacco Product	Conventional Cigarette	E-Cigarette
Total	464 (40.6%)	447 (39.1%)	278 (24.3%)
Academic Year (*n* = 1144)	Users *n* (%*)	Users *n* (%*)	Users *n* (%*)
First year (*n* = 253, 22.1%)	81 (32.0)	74 (29.2)	54 (21.3)
Second year (*n* = 281, 24.6%)	109 (38.8)	104 (37.0)	67 (23.8)
Third year (*n* = 262, 22.9%)	115 (43.9)	115 (43.9)	62 (23.7)
Fourth year (*n* = 245, 21.4%)	118 (48.2)	115 (46.9)	67 (27.5)
Fifth year** (*n* = 54, 4.7%)	20 (37.0)	20 (37.0)	11 (20.8)
Sixth year** (*n* = 49, 4.3%)	21 (42.9)	19 (38.8)	17 (34.7)

**p-value** ^**a**^	**0.008**	**0.001**	0.321
**Gender (n = 1144)**	**Users** n (%*)	**Users** n (%*)	**Users** n (%*)
Female (*n* = 634, 55.4%)	214 (33.8)	208 (32.8)	116 (18.3)
Male (*n* = 499, 43.6%)	244 (48.9)	234 (46.9)	158 (31.7)
Prefer not to say (*n* = 11, 1.0%)	6 (54.5)	5 (45.5)	4 (36.4)

**p-value** ^**a**^	**< 0.001**	**< 0.001**	**< 0.001**
Residential Status (*n* = 1144)	**Users** n (%*)	**Users** n (%*)	**Users** n (%*)
With family (*n* = 433, 37.8%)	182 (42.0)	175 (40.4)	125 (28.8)
In a dormitory (*n* = 489, 42.8%)	170 (34.8)	163 (33.3)	91 (18.6)
With a roommate (*n* = 140, 12.2%)	73 (52.1)	71 (50.7)	42 (30.2)
Living alone (*n* = 82, 7.2%)	39 (47.6)	38 (46.3)	20 (24.4)

**p-value** ^**a**^	**0.002**	**0.002**	**0.002**
Self-Perceived Economic Status (*n* = 1144)	**Users** n (%*)	**Users** n (%*)	**Users** n (%*)
Very low-Low (*n* = 133, 11.7%)	79 (59.4)	76 (57.1)	44 (33.3)
Moderate (*n* = 640, 55.9%)	238 (37.2)	231 (36.1)	142 (22.2)
Very high-High (*n* = 371, 32.4%)	147 (39.6)	140 (37.7)	92 (24.8)
**p-value** ^**a**^	**< 0.001**	**< 0.001**	**0.025**

*Row percentages are presented. To enhance clarity, solely the distribution of users is reported

**Fifth and sixth years are only applicable to the Faculty of Medicine

^a^ Pearson Chi-square test

**Table 2:** Distribution of tobacco product (including e-cigarette) use characteristics by faculty

Faculty	Total	Medicine	Engineering	Law	LHG	*p*-value
Tobacco Product Use Status (*n* = 1144)	*n* (%)*	*n* (%)*	*n* (%)*	*n* (%)*	*n* (%)*	
Currently using any tobacco product	464 (40.6)	111 (37.1)	81 (39.7)	124 (38.2)	148 (46.8)	0.018^a^
Non-user	610 (53.3)	177 (59.2)	111 (54.4)	179 (55.1)	143 (45.3)	
Former user	70 (6.1)	11 (3.7)	12 (5.9)	22 (6.8)	25 (7.9)	

Type of Tobacco Product Used **	n (%)*	n (%)*	n (%)*	n (%)*	n (%)*	
Cigarettes	447 (39.1)	105 (35.1)	80 (39.2)	121 (37.2)	141 (44.6)	0.089^a^
E-cigarettes	278 (24.3)	69 (23.2)	47 (23.0)	74 (22.8)	88 (27.8)	0.404^a^
Waterpipe(hookah)	89 (7.8)	17 (5.7)	22 (10.8)	23 (7.1)	27 (8.5)	0.181^a^
Roll-your-own tobacco	88 (7.7)	9 (3.0)	18 (8.8)	22 (6.8)	39 (12.3)	< 0.001^a^
Other products	16 (1.4)	2 (0.7)	3 (1.5)	4 (1.2)	7 (2.2)	0.449^b^

	**Median (1st–3rd Quartile)**	**Median (1st–3rd Quartile)**	**Median (1st–3rd Quartile)**	**Median (1st–3rd Quartile)**	**Median (1st–3rd Quartile)**	*p*-value
Cigarette Consumption (Pack-Year)(*n* = 431)	2 (1–4)	1.5 (0.5-3.0)^x^	2.3 (1.0–4.0)^x, y^	1.8 (0.6–4.5)^x, y^	2.3 (0.8-5.0)^y^	0.023^c^

Fagerstrom Nicotine Dependence Level (*n* = 431)	n (%)*	n (%)*	n (%)*	n (%)*	n (%)*	*p*-value
Very high-High	73 (16.9)	12 (12.8)	11 (14.3)	20 (16.8)	30 (21.3)	0.520^b^
Moderate	37 (8.6)	10 (10.6)	3 (3.9)	12 (10.1)	12 (8.5)	
Low-Very low	321 (74.5)	72 (76.6)	63 (81.8)	77 (73.1)	99 (70.2)	

LHG: Languages, History and Geography

* Column percentages are presented

** Multiple responses were allowed for type of tobacco product used

^a^ Chi-square test

^b^ Fisher-Freeman-Halton test

^c^ Kruskal–Wallis test. Post hoc pairwise comparisons were performed using Bonferroni correction. Groups sharing the same superscript letter (^x, y^) do not differ significantly

The distribution of participants’ use of any tobacco product, cigarette smoking, and e-cigarette use according to their academic year, gender, place of residence, and perceived economic status is presented in Table 1. Use of any tobacco product was found to be significantly associated with higher academic year, male gender, living alone or with a roommate, and very low/low perceived economic status (*p* = 0.008; *p* < 0.001; *p* = 0.002; *p* < 0.001, respectively). To further evaluate the factors independently associated with the use of any tobacco product, a multivariable logistic regression analysis was performed (Table 3). The results showed that faculty (*p* = 0.019), academic year (*p* = 0.006), gender (*p* < 0.001), and perceived economic status (*p* < 0.001) remained significantly associated with tobacco use, whereas age (*p* = 0.058) and residence status (*p* = 0.119) were not significant. Compared with students in Medicine, those in the Faculty of Languages, History and Geography had higher odds of tobacco use (Adj. OR = 1.81, 95% CI: 1.21–2.70, *p* = 0.004). Advancing academic year was also associated with greater odds of tobacco use overall (*p* = 0.006). Female students had lower odds compared with males (Adj. OR = 0.48, 95% CI: 0.37–0.62, *p* < 0.001). Likewise, students with moderate (Adj. OR = 0.46, 95% CI: 0.31–0.68, *p* < 0.001) or high/very high economic status (Adj. OR = 0.50, 95% CI: 0.33–0.77, *p* = 0.002) had lesser odds of tobacco use than those with low or very low economic status.

**Table 3:** Multivariate analysis of factors associated with any tobacco product (including e-cigarette) use

	Adj. OR	95% CI	*p* value
**Age**	0.96	0.92–1.00	0.058
**Faculty (ref. medicine)**			0.019
Engineering	1.21	0.78–1.87	0.405
Law	1.29	0.86–1.94	0.212
LHG	1.81	1.21–2.70	0.004
**Academic Year (ref. 1 st year)**			0.006
Second year	1.44	0.98–2.13	0.064
Third year	1.90	1.29–2.81	0.001
Fourth year	2.18	1.45–3.30	< 0.001
Fifth year	1.72	0.82–3.62	0.150
Sixth year	2.30	1.09–4.88	0.030
**Gender (ref. male)**			< 0.001
Female	0.48	0.37–0.62	< 0.001
Prefer not to say	1.05	0.30–3.60	0.942
**Perceived Economic Status (ref. low-very low)**			< 0.001
Moderate	0.46	0.31–0.68	< 0.001
Very high-High	0.50	0.33–0.77	0.002
**Residence Status (ref. with family)**			0.119
In a dormitory	0.78	0.59–1.04	0.092
With a roommate	1.19	0.79–1.78	0.416
Living alone	1.16	0.70–1.90	0.571

Hosmer and Lemeshow Test, *p* = 0,846

The distribution of tobacco users’ willingness to quit, quit attempts, awareness of available medical counselling support for tobacco dependence, and whether they were advised to quit by a healthcare provider—according to faculty—is presented in Table 4. A total of 72.8% (*n* = 326) of the students reported that they wanted to quit tobacco use. More than half of the students (52.5%, *n* = 234) had tried to quit at least once, with a median of two attempts. Among those who attempted to quit, only 2.6% (*n* = 6) did so with medical counselling support. Among students, 36.9% (*n* = 164) were aware of professional counselling services for tobacco dependence. Meanwhile, only 22.3% (*n* = 100) of tobacco users had been advised by a healthcare professional to quit within the past year. Among students, 63.9% (*n* = 174) who had ever considered quitting and 61.2% (*n* = 30) who planned to quit within the next month were unaware of how to access medical counselling support.

**Table 4:** Intentions to quit, previous attempts, and awareness of counselling services among tobacco-using university students, stratified by faculty

Faculty	Total	Medicine	Engineering	Law	LHG	*p*-value
Willingness to Quit Tobacco Use (*n* = 448), n(%)*						
Does not want to quit	122 (27.2)	22 (21.0)	14 (18.0)	40 (33.1)	46 (32.0)	0.103^a^
Wants to quit within the next month	49 (15.0)***	10 (9.5)	10 (12.8)	15 (12.4)	14 (9.7)	
Has wanted to quit at some point in life	277 (85.0)***	73 (69.5)	54 (69.2)	66 (54.5)	84 (58.3)	
**Previously Attempted to Quit Tobacco Use**(*n* =446), n (%)*	234 (52.5)	60 (57.1)	38 (48.7)	59 (49.6)	77 (53.5)	0.611^a^
Aware of Medical Counselling Support for Tobacco Dependence ** (***n* =444), n (%)**	164 (36.9)	45 (42.5)	30 (39.5)	38 (31.7)	51 (35.9)	0.378^a^
Was Advised to Quit by a Healthcare Provider During a Medical Visit in the Last 12 Months **(***n* =448), n (%)**	100 (22.3)	23 (21.9)	18 (23.1)	25 (20.7)	34 (23.6)	0.947^a^

LHG = Faculty of Languages, History and Geography

*Column percentages are presented

**Only positive responses are reported

***Distribution is calculated among those who expressed a willingness to quit

^a^ Chi-square test

Among tobacco-using students, 38.1% (*n* = 171) had never tried e-cigarettes, 7.6% (*n* = 34) used them regularly, and 54.3% (*n* = 244) used them occasionally. Overall, 70.5% (*n* = 318) had a close contact who vapes. The main reasons for e-cigarette use included enjoying flavours (60.1%, *n* = 167), curiosity (45.3%, *n* = 126), smoking cessation (19.4%, *n* = 54), recommendations from others (8.6%, *n* = 24), and other factors such as lower cost and lack of odor (1.8%, *n* = 5).

Among tobacco users, 81.5% correctly identified that e-cigarettes may contain harmful substances beyond nicotine, while only 46.1% correctly answered that e-cigarette liquid can be absorbed through the skin and cause poisoning. The full response distribution is shown in Table 5. The median number of correct answers out of 14 e-cigarette-related statements was 9 (1st – 3rd Quartiles: 7–12).

**Table 5:** Reasons for e-cigarette use and distribution of responses to knowledge statements on e-cigarettes

Reasons for Using E-Cigarettes (*n* = 278)	*n* (%*)
Flavour and taste	167 (60.1)
Curiosity	126 (45.3)
To quit smoking	54 (19.4)
Upon recommendation	24 (8.6)
Other**	5 (1.8)

Knowledge Statements on E-Cigarettes (*n* = 453)	n (%)***
*E-cigarettes may contain harmful substances in addition to nicotine*	369 (81.5)
*E-cigarettes do not cause addiction like other tobacco products.*	356 (78.6)
*Carcinogenic substances found in other tobacco products are not present in e-cigarettes*	335 (74.0)
*Some e-cigarette batteries have caused fires and explosions resulting in serious injuries.*	333 (73.5)
*Nicotine can harm brain development in young adults*,* and this risk continues into the mid-20s.*	331 (73.1)
*Even short-term use of e-cigarettes can cause severe lung damage and respiratory failure*,* which may be fatal.*	306 (67.5)
*E-cigarette vapor does not harm others like other tobacco products do.*	299 (66.0)
*E-cigarettes can be used in indoor environments.*	288 (63.6)
*E-cigarettes contain ultrafine particles that can be inhaled deep into the lungs.*	280 (61.8)
*E-cigarettes are effective as a smoking cessation method.*	273 (60.3)
*E-cigarettes are not considered tobacco products.*	268 (59.2)
*Some e-cigarette liquids have been found to contain metals such as tin*,* lead*,* nickel*,* chromium*,* manganese and arsenic.*	215 (47.5)
*Some e-cigarettes marketed as nicotine-free have been found to contain nicotine.*	210 (46.4)
*E-cigarette liquid can be absorbed through the skin and may cause poisoning.*	209 (46.1)
	Median (1st–3rd Quartile)
Number of Correct Responses	9 (7–12)

* Multiple responses allowed

** One student cited affordability; four students cited lack of odor in indoor environments

*** Only the number and percentage of participants who correctly identified the accuracy of each statement are reported

## Discussion

This study, which aimed to examine the prevalence of cigarette and e-cigarette use, willingness to quit, and related beliefs among university students, revealed that approximately 4 out of every 10 students used a tobacco product, multi-product tobacco use was common, and e-cigarettes were increasingly preferred by young people. The higher prevalence of tobacco product use found in this study compared to 2016 GATS may be explained by several factors. The growing popularity of e-cigarettes among young people in recent years, increased levels of stress, anxiety, and depression among students due to the COVID-19 pandemic and economic difficulties, and the stronger social acceptance of anti-smoking policies in the past are all possible contributors to this increase [10, 17, 18]. Similarly, the 2023 National Survey on Drug Use and Health, conducted by the U.S. Substance Abuse and Mental Health Services Administration (SAMHSA), reported that 40.9% of 18–25-year-olds had used a tobacco product, including e-cigarettes, within the past year [19]. In contrast, the same agency’s 2016 survey did not include questions about e-cigarettes but reported a 23.5% prevalence of cigarette smoking in the 18–25 age group [20]. Based on these findings, it is reasonable to predict that a future national survey in Turkey covering e-cigarettes and heated tobacco products would likely show an increased prevalence in this age group. In the present study, it was found that approximately 1 in 4 students had been exposed to e-cigarettes. However, in a study conducted in 2015, when these products were not yet widespread in Turkey, only 9 out of 1241 university students reported using e-cigarettes; half of the participants said they had never heard of e-cigarettes, and 73% had never seen one. In contrast, the present study shows that 70.5% of tobacco users had at least one acquaintance who uses e-cigarettes [21]. The fact that e-cigarette use has become so widespread in such a short period—in a country where their sale is not even legally permitted—suggests that interventions in this area must be urgently developed and expanded.

According to the 2016 Global Adult Tobacco Survey (GATS), the prevalence of cigarette smoking among young people aged 15–24 in Turkey was 40.9% in males and 22.4% in females [13]. In the present study, however, the prevalence of tobacco use among female students was considerably higher than these earlier estimates, suggesting a potential increase in use among women over the past decade [22]. This rise may be related to changing social roles, higher levels of stress, targeted marketing strategies of the tobacco industry, and perceptions concerning weight control. The study also found that tobacco use became more common as students advanced through academic years, which may be explained by increasing academic workload, examination stress, and growing concerns about the future as graduation approaches. Qualitative research likewise shows that students often describe smoking as a means of maintaining concentration or obtaining short-term relief in stressful situations [23].

In addition to the increasing prevalence of tobacco product use among university students, the concurrent use of multiple tobacco products is another issue that warrants attention. In this study, the dual use of cigarettes and e-cigarettes was particularly notable, as almost all students who used e-cigarettes also smoked cigarettes. A study conducted in China in 2021 with approximately 9,000 university students found that 29.8% of participants used either cigarettes or e-cigarettes, with 16.7% using only e-cigarettes, 35.0% using only cigarettes, and 48.3% using both products simultaneously [24]. According to Centers for Disease Control and Prevention (CDC) data, 3 out of every 10 adults who use e-cigarettes also smoke cigarettes [25]. Similarly, a cohort study found that younger age was associated with multi-product tobacco use [26].

On the other hand, an encouraging finding of this study is that the majority of cigarette-smoking students were found to have “low” or “very low” levels of nicotine dependence. Similar results have been reported in various studies conducted at different universities, indicating that students typically have low levels of nicotine addiction [27–29]. The fact that most participants had low nicotine dependence and relatively low cigarette consumption in terms of pack-years may suggest that they had only recently initiated tobacco use. A large-scale study examining trends in smoking initiation among individuals aged 18–23 in the United States between 2002 and 2018 reported that the proportion of those who started smoking within this age group more than doubled, increasing from 20.6% to 42.6% [30]. These findings suggest that this age period represents a high-risk window globally for initiating tobacco use. It is well established that the earlier a person starts smoking, the higher the risk of developing strong physiological dependence on nicotine [31]. Therefore, the generally low levels of nicotine dependence observed in university-based studies and the fact that the average age of initiation corresponds to this period further emphasize the importance of early intervention and prevention strategies, both for individual health and for reducing the global burden of disease.

Among the WHO MPOWER (Monitor, Protect, Offer, Warn, Enforce, Raise), strategies, the recommendation to quit tobacco use is especially valuable in this context. In this study, it was found that only one in five students who used tobacco products had been advised to quit during a medical facility visit within the past year. Comparable findings have been reported elsewhere, with 15% in India [32] and 22% in Italy [33] receiving such advice. Even brief and simple advice from physicians can significantly increase the likelihood of successful quitting and sustained abstinence [34]. Although university students may not routinely undergo medical check-ups, in Turkey some faculties (e.g., health sciences) require periodic examinations, and campus-based health clinics that serve only students are widely available. These encounters provide a critical opportunity to integrate routine screening for tobacco use and brief cessation advice into student healthcare services.

The study revealed that nearly three out of four students expressed a desire to overcome tobacco addiction. When combined with low levels of nicotine dependence, this presents a significant opportunity for intervention. However, occasional or light smokers may face unique challenges in quitting, such as lower perceived addiction, reduced motivation to seek support, or underestimation of health risks. These barriers may hinder cessation despite low physiological dependence [35]. These students should not be left unsupported, and they must be actively supported by universities and health authorities. It is not sufficient to merely offer medical counselling support; such services must also be actively promoted and made visible. Only 36.9% of tobacco-using students knew how to access medical counselling support. Notably, even among medical students, who are expected to have higher health literacy than their peers, more than half were unaware of the available medical counselling support for treating tobacco dependence.

Although students demonstrated a strong inclination to quit smoking, the process often involved repeated unsuccessful attempts. In this study, it was found that half of the students had attempted to quit tobacco product use at least twice. However, despite this positive inclination, nearly all quit attempts occurred without access to medical counselling support. Similar patterns have been observed in other countries as well. A study conducted in five European countries among university students found that half of the participants intended to quit smoking in the near future, yet 87.5% had not sought professional support for cessation [36]. In Switzerland, a study involving 2574 participants that examined the experiences of healthcare personnel with smoking cessation revealed that individuals who had two failed quit attempts were 2.58 times more likely to fail again. While the chances of successfully quitting were higher during the first or second attempt, the likelihood of quitting without support declined significantly after a third attempt [37]. These findings suggest that students’ knowledge gaps should be addressed not only regarding the harms of tobacco products, but also concerning effective methods used in the quitting process, such as pharmacological treatments and behavioural therapies.

In this study, the least well-known statements regarding e-cigarettes concerned the presence of heavy metals, the possibility that supposedly nicotine-free products may in fact contain nicotine, the risk of absorption through the skin, and the classification of e-cigarettes as tobacco products. These findings highlight persistent knowledge gaps, consistent with previous studies in Turkey and internationally, which have shown that young adults often underestimate the harms of e-cigarettes or misidentify their contents [16, 38–40]. Misconceptions were also evident in perceptions of harm: approximately four in ten tobacco-using students believed that e-cigarettes do not harm others, and a similar proportion were unaware of indoor use restrictions. The main motivations for e-cigarette use in this study—flavour, curiosity, and, to a lesser extent, an intention to quit smoking—reflect patterns observed elsewhere, where flavours and curiosity are consistently reported as primary drivers of experimentation. However, robust evidence does not support the effectiveness of e-cigarettes as a cessation tool, and students who underestimate the risks may perceive occasional use as harmless. Notably, nearly one-third of tobacco-using students were unaware that even short-term use of e-cigarettes can cause lung damage and increase the risk of respiratory failure. The persistence of these misconceptions, despite the legal ban on sales in Turkey, underscores the urgent need for targeted education and stronger enforcement measures to prevent further uptake among university students.

Our findings underline the need for comprehensive, campus-based interventions. Expanding smoke-free campus policies, ensuring access to professional and psychological counselling, and training physicians in student health clinics to provide routine cessation advice are critical measures. Awareness should be strengthened through campus events, posters, social media campaigns, and informational booths. In addition, digital support programmes—such as mobile applications, SMS-based systems, and peer-led groups—have shown promise in increasing cessation rates, particularly among young adults, and should be actively implemented. Incentive-based activities (e.g., small rewards) can help sustain participation in cessation programmes over time [41]. Taken together, these strategies would not only increase awareness but also support behavioural change by addressing both structural and individual-level factors.

While the findings obtained are meaningful, the study has several methodological limitations. Due to legal constraints, it was not possible to use random sampling, and student participation within classrooms was based on voluntary consent. Another limitation was that not all departments of the Faculty of Engineering granted administrative approval. However, it was not assumed that there would be significant differences in tobacco use habits between departments that agreed to participate and those that did not respond. Students were asked about their current use of tobacco products without a specific timeframe, which limited the ability to make a precise distinction regarding current use. However, the fact that all data were collected face-to-face by a single researcher ensured consistency and standardization in data collection. Although the study was conducted at Ankara, the university’s location in the capital city and its status as a well-established institution attracting students from various geographical regions enhanced the generalizability of the results. Moreover, by distributing informational leaflets after the survey—containing accurate information about e-cigarettes, the benefits of quitting tobacco, and available medical counselling support—the study not only aimed to assess the current situation but also contributed to raising awareness among students, which represents a significant added value.

## Conclusion

This study revealed that tobacco use, including e-cigarettes, remains highly prevalent among university students in Turkey, with multi-product use being common. While most students demonstrated low nicotine dependence and a willingness to quit, significant gaps in knowledge—particularly regarding e-cigarettes—and limited awareness of cessation support services persist. Addressing these gaps requires comprehensive, campus-based strategies that combine smoke-free policies, digital and peer-led cessation support, educational initiatives to correct misconceptions, and access to professional counselling within university health services. At a broader level, sustainable success in tobacco control also depends on nationally restructured policies supported by current data, with healthcare professionals playing a central role in routinely delivering cessation counselling and guidance.

## Supplementary Information

Supplementary Material 1

## Data Availability

The datasets used and/or analysed during the current study are available from the corresponding author on reasonable request.

## Abbreviations

WHO: World Health Organization
E-cigarette: Electronic cigarette
GATS: Global Adult Tobacco Survey
SAMSHA: Substance Abuse and Mental Health Services Administration
CDC: Centers for Disease Control and Prevention
