# Supplementary material for: Understanding tobacco and e-cigarette use among university students: a cross-sectional study exploring nicotine dependence, quit intentions, and awareness of cessation support services
Source: BMC Public Health. 2025 Oct 27;25:3614. doi: 10.1186/s12889-025-24911-6 (PMC12560475; doi:10.1186/s12889-025-24911-6)
Supplement: Supplementary file 1 — Supplementary Material 1 [file 12889_2025_24911_MOESM1_ESM.pdf]

Questionnaire Form (English)

Please write your faculty and grade: ____________________

1.Please write your year of birth: ____________________

2.Please mark the option that best describes your gender:
- [ ] Female
- [ ] Male
- [ ] Prefer not to say

3.Please select the option that best describes your current status of residence.
- [ ] With family
- [ ] In a dormitory
- [ ] With a roommate
- [ ] Living alone
- [ ] Other, please specify: ______________________

4. Please select the option that best describes your economic status.
- [ ] Very low
- [ ] Low
- [ ] Moderate
- [ ] High
- [ ] Very high

5. Which of the following do you use? (You may select more than one option.)

- [ ] I have never used any tobacco products in my life.
- [ ] I used to use tobacco products and quit with medical support (How long ago? _______)
- [ ] I used to use tobacco products and quit on my own (How long ago? _______)
- [ ] Cigarette
- [ ] Waterpipe(hookah)
- [ ] Roll-your-own tobacco
- [ ] Electronic cigarette
- [ ] Other products, please specify: ___________________

6.What is your opinion about the smoking ban in indoor areas? Please rate from 1 to 10 (1 = strongly disagree, 10 = strongly agree).

1 2 3 4 5 6 7 8 9 10

<u>Participants who do not currently use tobacco products may end the questionnaire here. Thank you for your participation.</u>

7. How long have you been using tobacco products? Please specify: ___________________

8. How much tobacco do you use per day? (For example: 10 cigarettes, 20 puffs of e-cigarette, 1 hookah session, etc.) Please specify ___________________

9. Do you want to quit using tobacco products?
[ ] Yes, I want to quit at some point in my life.
[ ] Yes, I want to quit within the next month.
[ ] No.

10. Have you ever attempted to quit using tobacco products?
[ ] Yes, I have attempted to quit.
How many times have you attempted? Please specify: ___________________
[ ] No, I have never attempted to quit. **(If you select this option, please proceed to question 12.)**

11. Have you ever received medical counseling support to quit using tobacco products
[ ] Yes, I have received medical counseling support. (Please briefly explain)
____
____
[ ] No, I have attempted to quit on my own.

12.Do you have information about the ways in which you can get medical counseling support to quit using tobacco products?
[ ] Yes (Please briefly explain)
____
____
[ ] No

13. Have you been advised to quit tobacco product use by a healthcare provider during a medical visit in the last 12 months?
[ ] Yes
[ ] No

14. Please select the option that best corresponds to your experience with electronic cigarette use.
[ ] I have never used electronic cigarettes. **(If you select this option, please proceed to question 17.)**
[ ] Yes, I use them but not regularly.
[ ] Yes, I currently use them regularly. Please write your age at the time you started___________)

15.What are the reasons using electronic cigarettes? (Multiple responses may be selected.)
[ ] Curiosity
[ ] Upon recommendation
[ ] Because they have good flavor and taste
[ ] To quit smoking
[ ] Other, Please write: ___________________

16. Where did do you get electronic cigarettes? (You may select more than one option.)
[ ] Internet
[ ] Store/Shop
[ ] Through a friend, relative, or acquaintance
[ ]Other, please write: ______________________

17.Do you have any relatives around you who use electronic cigarettes?

[ ] Yes

[ ] No

18. Please select the statement that you believe is correct regarding electronic cigarettes (e-cigarettes).

	True	False	No opinion
Nicotine can harm brain development in young adults, and this risk continues into the mid-20s.			
E-cigarettes do not cause addiction like other tobacco products.			
Even short-term use of e-cigarettes can cause severe lung damage and respiratory failure, which may be fatal.			
E-cigarettes are effective as a smoking cessation method.			
E-cigarette vapor does not harm others like other tobacco products do.			
E-cigarettes can be used in indoor environments.			
Some e-cigarettes marketed as nicotine-free have been found to contain nicotine.			
E-cigarettes contain ultrafine particles that can be inhaled deep into the lungs.			
Carcinogenic substances found in other tobacco products are not present in e-cigarettes.			
E-cigarettes may contain harmful substances in addition to nicotine			
Some e-cigarette liquids have been found to contain metals such as tin, lead, nickel, chromium, manganese and arsenic.			
E-cigarettes are not considered tobacco products.			
Some e-cigarette batteries have caused fires and explosions resulting in serious injuries.			
E-cigarette liquid can be absorbed through the skin and may cause poisoning.			

19. Please select the option that you find most appropriate.

How soon after waking do you smoke first cigarette?			
[ ] Within 5 minutes	[ ] 5-30 minutes	[ ] 31-60 minutes	[ ] After 60 minutes
Do you find it difficult not to smoke in places where it is not allowed (For example: school, hospital, cinema, bus, etc)			
[ ] Yes		[ ] No	
Which cigarette would you hate to give up most?			
[ ] Your first cigarette after you wake up		[ ] Any other cigarette during the day	
How many cigarette per day do you smoke?			
[ ] 10 or fewer cigarettes	[ ] 11 to 20 cigarettes	[ ] 21 to 30 cigarettes	[ ] 31 or more cigarettes
Do you smoke more during the first hours after you wake up than during the rest of the day?			
[ ] Yes		[ ] No	
If you are so sick that you have to stay in bed most of the day, do you still smoke?			
[ ] Yes		[ ] No	

The questionnaire is complete. Thank you for your participation.
